# 3q26 Amplifications in Cervical Squamous Carcinomas

**DOI:** 10.3390/curroncol28040251

**Published:** 2021-07-29

**Authors:** Ioannis A. Voutsadakis

**Affiliations:** 1 Algoma District Cancer Program, Sault Area Hospital, Sault Ste. Marie, ON P6B 0A8, Canada; ivoutsadakis@nosm.ca; 2 Section of Internal Medicine, Division of Clinical Sciences, Northern Ontario School of Medicine, Sudbury, ON P3E 2C6, Canada

**Keywords:** amplification, molecular genetics, uterine cervix, squamous carcinoma

## Abstract

Background: Squamous carcinomas of the uterine cervix often carry mutations of the gene encoding for the catalytic sub-unit of kinase PI3K, *PIK3CA*. The locus of this gene at chromosome 3q26 and neighboring loci are also commonly amplified. The landscape of 3q26-amplified cases have not been previously characterized in detail in cervical cancer. Methods: Published genomic data and associated clinical data from TCGA cervical cancer cohort were analyzed at cBioportal for amplifications in genes at 3q26. The clinical and molecular characteristics of the group of patients with 3q26 amplifications was compared with the group without 3q26 amplifications. Comparative prevalence of amplification and expression of genes at 3q26 in amplified squamous cervical cancer cases were surveyed as well as 3q26 amplifications in cervical cancer cell line databases. Results: Amplification of 3q26 locus is a prevalent molecular lesion in cervical squamous cell carcinomas encountered in about 15% of cases in TCGA cohort of 247 patients. Cancer-related genes commonly amplified from 3q26 include *PIK3CA*, *TBL1XR1*, *DCUN1D1*, *SOX2,* *MECOM*, *PRKCI*, and TERC. Amplified cases do not completely overlap with *PIK3CA* mutant cases. Differences exist between 3q26-amplified and non-amplified carcinomas in the frequency of mutations and frequency of other amplifications. Most commonly over-expressed genes in 3q26 amplified cases include *PIK3CA*, *TBL1XR1*, *DCUN1D1*, and less commonly *SOX2* and *PRKCI*. Conclusion: The subset of squamous cervical carcinomas with 3q26 amplifications is not overlapping with cancers carrying *PIK3CA* mutations and contains, besides PIK3CA, other cancer-associated genes that are over-expressed at the mRNA level, including *TBL1XR1* and *DCUN1D1*. *DCUN1D1*, a regulator of SCF ubiquitin ligase activity, may be a relevant pathogenic player given the importance of ubiquitination and the proteasome in the disease. These observations could form the basis for therapeutic exploitation in this subset of squamous cervical carcinomas.

## 1. Introduction

Squamous cervical cancers are viral associated carcinomas in their majority and represent a significant health problem worldwide, with over 600,000 cases diagnosed and 340,000 deaths in 2020 [1]. High-risk types of the human papilloma virus (HPV) are prevalent and cause the disease by integrating their genome to cervical cells, promoting immortalization [2]. It is hoped that the recent introduction of HPV polyvalent vaccines will decrease the burden of the disease, supplementing early detection with screening programs and effective treatments of localized disease with chemo-radiotherapy [3,4]. However, despite the introduction of new effective therapies, including antiangiogenic and immunotherapies, metastatic disease remains incurable, and an unmet need of additional effective treatments exist [5].

The molecular landscape of squamous cervical carcinomas has been elucidated with the recent publication of genomic studies by the Cancer Genome Atlas (TCGA) initiative and others [6]. A main difference of the pathogenesis of squamous cervical carcinomas from several other cancers is that they rarely carry mutations in the major tumor suppressor p53. Instead, the function of p53 is neutralized by virally assisted ubiquitination and proteasome degradation. The HPV E6 viral protein is implicated in p53 degradation [2]. Another HPV viral protein, E7, neutralizes the function of tumor suppressor Rb (retinoblastoma). Other molecular lesions assist in neoplastic transformation, with oncogenic mutations in the catalytic sub-unit of kinase PI3K (phosphatidylinositol-4,5-bisphosphate 3-kinase) being the most prevalent molecular lesion encountered in about a quarter of cervical carcinomas [7]. These mutations activate a signal transduction pathway with several downstream effectors, promoting cell proliferation and apoptosis inhibition [8]. PI3K mutations are prevalent in other cancers and have been targeted with specific inhibitors that are currently used in metastatic breast cancers carrying PI3K mutations [9,10]. In addition, PI3K inhibitors with specificity for the δ subunit are used in hematologic malignancies [11]. In squamous cervical carcinomas, PI3K inhibitors have not yet been introduced in clinical practice. Besides mutations, amplifications of the 3q26 locus, where the *PIK3CA* gene encoding for the catalytic alpha sub-unit of PI3K resides, are often observed in squamous cervical carcinomas. The locus harbors several cancer-related genes that could contribute to the pathogenesis of the disease. Amplifications of the long arm of chromosome 3 had been identified even before the genomic era as important in squamous epithelial carcinogenesis [12]. The landscape of 3q26-amplified squamous cervical cancers is discussed in this investigation that explores published genomic data with the aim to derive an understanding of cases with 3q26 amplifications that could assist in future targeted drug development.

## 2. Methods

The Cancer Genome Atlas (TCGA) study of cervical cancer was surveyed to identify patients with or without 3q26 amplifications. The cohort consists of a total of 297 patients among whom 46 patients have adenocarcinoma and 251 patients have squamous cervical carcinoma [6]. Adenocarcinoma patients were excluded from the current analysis, which included exclusively the 251 patients with squamous uterine cervix carcinoma. The groups with or without 3q26 amplifications were compared to discover differences in relevant clinical and genomic characteristics of interest. Comparisons with 3q26 amplifications in other squamous carcinomas were derived through the respective cohorts of TCGA for these carcinomas [13,14,15,16].

The analysis was performed in the online cBioPortal for Cancer Genomics Portal (cBioportal, http://www.cbioportal.org, accessed on 22 May 2021), a genomics site initially developed by Memorial Sloan Kettering Cancer Center (MSKCC) and currently maintained by MSKCC in collaboration with others [17,18]. cBioPortal is an open, user-friendly source which allows investigators to interrogate the database of included genomic studies for any gene of interest. The database includes data on mutations, copy number alterations (CNAs), and mRNA expression as well as correlative clinical data from TCGA and other groups [17]. TCGA uses the GISTIC (Genomic Identification of Significant Targets in Cancer) algorithm for the analysis of CNAs. According to the GISTIC algorithm, a score of 2 or above denotes putative amplification of a gene, while a score of −2 and below denotes putative deep deletion [19]. TCGA calculates an aneuploidy score (AS) as a measure of chromosomal instability in the samples included [20]. AS is derived by summing the number of chromosome arms in each sample that have copy number alterations (gains or losses). A chromosome arm is considered copy-number altered, either gained or lost, if somatic copy-number alterations are present in more than 80% of the length of the arm as calculated by the ABSOLUTE algorithm from Affymetrix 6.0 SNP arrays [21]. Chromosomal arms with somatic copy-number alterations in 20% to 80% of the arm length are not called, and chromosomal arms with somatic copy-number alterations in less than 20% of the arm length are considered not altered (diploid). For the mRNA analysis, the RSEM algorithm was used for normalization of mRNA expression [22].

Putative pathogenic implications of alterations in cancer genes of interest were derived from the OncoKB knowledgebase, which classifies listed genes as cancer-related, oncogenes, or tumor suppressors [23].

The Genomics of Drug Sensitivity in Cancer (GDSC, www.cancerrxgene.org, accessed on 22 May 2021) is an open access database maintained by Sanger Institute and Broad Institute, Massachusetts General Hospital Cancer Center, which contains information of array experiments testing drug sensitivities of cancer cell lines [24]. The database was queried for drug sensitivity of cell lines derived from cervical cancer patients with and without 3q26 amplification. In addition, dependencies of cervical cell lines to specific genes was obtained from the DepMap portal that contains data from CRISPR array screens and RNA interference array screens performed on cell lines from the Cancer Cell Line Encyclopedia (CCLE) [25,26].

Databases used in this study were queried on 16 February through 6 May of 2021. Statistical comparisons of categorical and continuous data were carried with the Fisher’s exact test or the χ^2^ test and the *t*-test. Prognosis of the groups of squamous cervical cancer patients with or without 3q26 amplifications was evaluated by construction of Kaplan–Meier curves. The log rank test was used to compare Kaplan–Meier survival curves. All statistical comparisons were considered significant if *p* < 0.05.

## 3. Results

**Figure 1:**
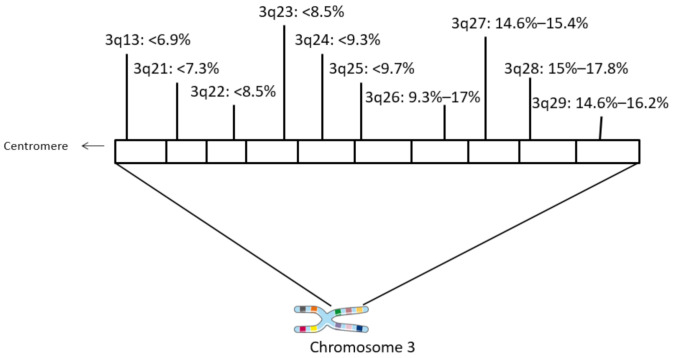
Schematic of chromosome 3q with frequencies of amplification in different loci in squamous cervical carcinomas. Data are from TCGA cervical cohort.

The locus 3q26.32 harboring *PIK3CA* and neighboring loci at the long arm of chromosome 3 are the most commonly amplified chromosome regions in squamous carcinomas of the uterine cervix (Figure 1). Loci of 3q, situated more centromeric to 3q26 as well as the centromeric part of 3q26, are amplified with a lower frequency. Amplification of *PIK3CA* is observed in 15.8% of samples in the cervical carcinoma cohort of TCGA. In 87% to 95% of these cases, the amplification extends to encompass regions beyond 3q26.32 and include surrounding 3q26 loci and neighboring 3q27–3q29 loci (Table 1). These loci are amplified in a similar number of cases as 3q26.32 and harbor several genes that are listed as cancer associated in the OncoKB database. In addition, amplicons at 3q26 to 3q29 are commonly encountered in squamous cell carcinomas of various locations besides the uterine cervix and are in fact even more prevalent in squamous cell carcinomas of the lung and the esophagus (Table 2). In contrast, adenocarcinomas of the same locations display a lower prevalence of amplifications of genes in 3q26–29, suggesting that these molecular lesions are characteristic of squamous carcinogenesis.

**Figure 2:**
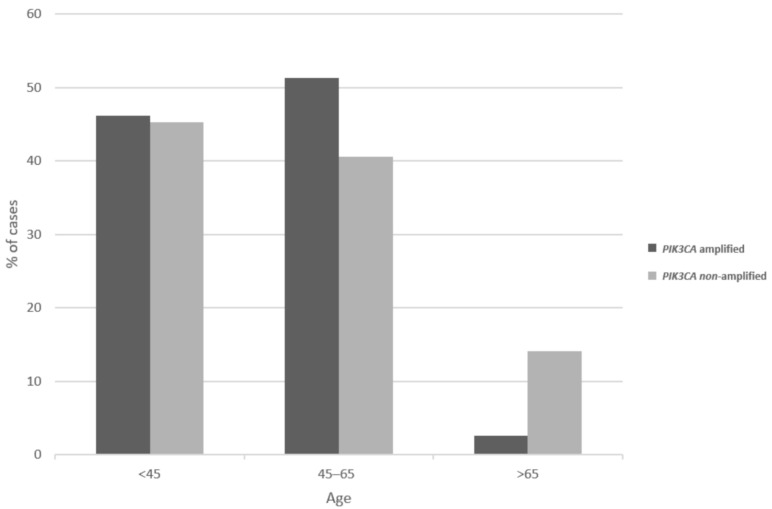
Distribution of age at presentation in squamous cervical carcinomas with and without 3q26 amplifications. Data are derived from the TCGA cervical cancer cohort.

**Figure 3:**
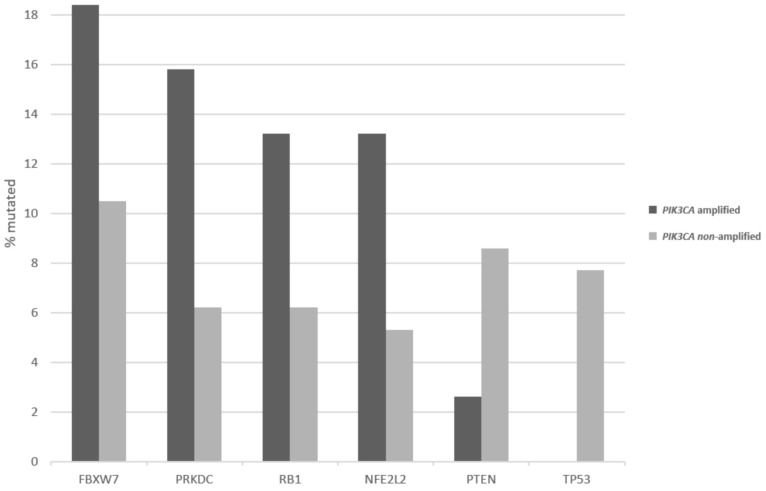
Percentage of mutations in the most common squamous cervical cancer-associated genes in cervical cancers with and without 3q26 amplifications. Data are derived from the TCGA cervical cancer cohort.

**Figure 4:**
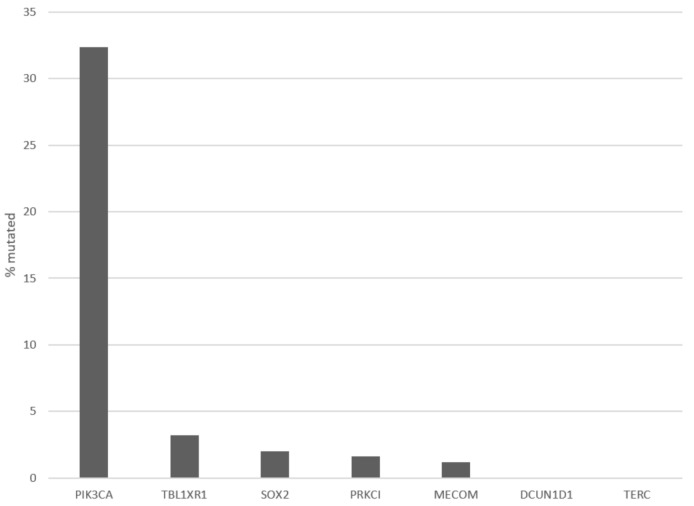
Frequency of mutations of cancer associated genes from chromosome arm 3q26 in the TCGA cervical cancer cohort.

Patients with 3q26 amplifications (as defined by amplifications of *PIK3CA* gene) present at an older age (65 years old and older) in 14.2% of cases, while patients without the amplification present only rarely (2.6% of cases) at an advanced age (Figure 2, χ^2^ *p* = 0.04). Among frequently mutated genes in squamous cervical cancers, several display higher prevalence of mutations in 3q26-amplified cervical cancers, as defined by amplifications of *PIK3CA* gene (Figure 3). These include the gene encoding for the ubiquitin ligase FBXW7, which is mutated in 18.4% of 3q26-amplified and in 10.5% of non-amplified cancers, and the gene encoding for the catalytic sub-unit of DNA-activated protein kinase PRKDC, which is mutated in 15.8% of 3q26-amplified and in 6.2% of non-amplified cancers (Figure 3). In addition, the gene encoding for the tumor suppressor RB1, which is mutated in 13.2% of 3q26-amplified and in 6.2% of non-amplified cancers, shows higher mutation prevalence in 3q26-amplified cancers (Figure 3). In contrast, tumor suppressors *PTEN* and *TP53* are more frequently mutated in 3q26-non-amplified cervical cancers. *PTEN* is mutated in 2.6% of amplified cancers and in 8.6% of non-amplified cancers, while *TP53* shows no mutations in 3q26-amplified cancers and is mutated in 7.7% of non-amplified cervical cancers. Among the 7 putative cancer-associated genes at 3q26 listed in OncoKB database, only *PIK3CA* is frequently mutated in squamous cervical carcinomas in 32.4% of cases, while the other genes are rarely mutated in 3.2% or fewer cases. *DCUN1D1* and *TERC* display no mutations in any of the 251 cases of the squamous cervical carcinoma (TCGA cohort, Figure 4). However, *PIK3CA* mutations are equally distributed between the 3q26-amplified and non-amplified groups (26.3% of amplified cases and 27.3% of non-amplified cases have a *PIK3CA* mutation, χ^2^ *p* = 0.9).

**Figure 5:**
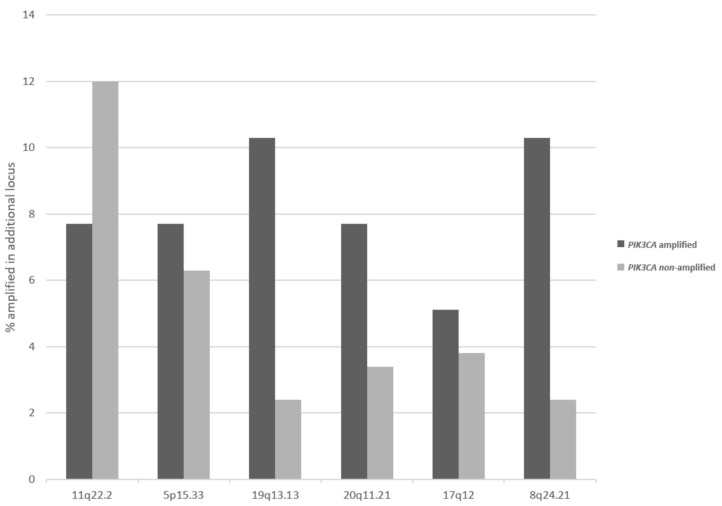
Frequency of amplifications in other chromosomal loci in cervical cancers with and without 3q26 amplifications. Data are derived from the TCGA cervical cancer cohort.

Besides loci at 3q, other regions that are commonly amplified in squamous cervical carcinomas display differences in prevalence of their amplification in 3q26-amplified and non-amplified cancers. The second most commonly amplified locus in cervical cancers, 11q22.2 is more often amplified in 3q26-non-amplified cancers (12%) than in 3q26.32-amplified cancers (7.7%), but this difference does not reach statistical significance (Figure 5). In contrast, several other loci are more commonly amplified in 3q26-amplified cervical carcinomas compared with non-amplified ones, and this difference is statistically significant for locus 19q13.2, which harbors the genes for kinases AKT2 and MAP3K10, and locus 8q24.21, which harbors the gene for transcription factor POU5F1B (Fisher’s exact test *p* = 0.03, Figure 5).

**Figure 6:**
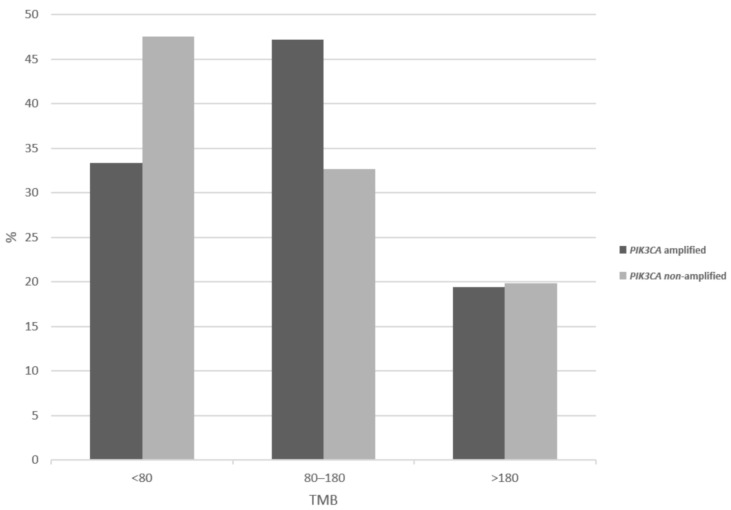
Percentage of total mutation burden (TMB) levels in cervical cancer patients with and without 3q26 amplifications.

Tumor mutation burden (TMB) evaluation discloses that *PIK3CA* amplified cervical cancers show less frequently than non-amplified cancers a low TMB of less than 80 (33.3% versus 47.5%), while amplified cancers have a higher prevalence of intermediate TMB of 80 to 180 (Figure 6). However, the two groups show no significant differences in the high-TMB category prevalence (19.4% of amplified versus 19.8% of non-amplified cancers show a TMB above 180, χ^2^ *p* = 0.2), which is most relevant for response to immunotherapy with immune checkpoint inhibitors. Regarding chromosomal instability as evaluated by the aneuploidy score (AS), 3q26-non-amplified cancers are slightly more frequently chromosomally stable (AS below 4 in 20.5% versus 15.3% in amplified cancers, χ^2^ *p* = 0.2, not shown).

At the mRNA level, cancer-associated genes at 3q26 are expressed in variable levels in squamous cervical carcinomas. Over-expression of *PIK3CA* and *TBL1XR1* and of neighboring gene *DCUN1D1* at 3q26.33 occur in 64.1% (for the two former) and 79.5% (for the latter) of 3q26-amplified cases, as defined by a z score of 2 or above compared with diploid samples (Table 3). *SOX2* and *PRKCI* display lower levels of mRNA over-expression in 3q26-amplified squamous cervical cancers, while *MECOM* and *TERC* show no over-expression.

Survival analysis showed that cervical carcinomas with and without 3q26 amplifications had similar progression-free and overall survival (log rank test *p* = 0.46 and 0.69, respectively, not shown).

In the Genomics of Drug Sensitivity in Cancer (GDSC) database, part of the 3q26 locus, including SOX2 and DCUN1D1 genes, is identified as a recurrent copy-number alteration in the pan-cancer analysis (feature cnaPANCAN246 in the database). However, among the 13 cell lines listed in the cervical cancer cohort (some being with adenocarcinoma histology), none was positive for the amplification, suggesting that the 3q26 amplification in cervical squamous cell carcinomas is not well depicted in the available in vitro cell line models of the disease. In addition, several cell line models do possess *TP53* mutations, which are rare in patients with cervical squamous cell cancers, as the tumor suppressor is neutralized by an alternative mechanism through E6-AP-mediated ubiquitination and proteasome degradation. Related to this, ubiquitination-related proteins arise as the most important dependencies in the squamous cervix cohort of cell lines as assessed by CRISPR knockdown in the dependencies map (DepMap) portal of the Broad Institute. Among the top eight genes whose knockdown significantly affects survival of cervical cell lines, six genes are ubiquitin ligases, including E6-AP (the cellular target for activation by HPV E6 protein, alternatively called UBE3A) and SKP2 (Table 4).

## 4. Discussion

Copy-number alterations, amplifications, and deep deletions are observed in all cancers, and type-specific, recurrent amplifications have been shown in various cancers [24]. Each tumor contains a median of 19 recurrently aberrant copy-number segments, with a median number of 15 genes in amplified segments and a median of one gene in homozygous deleted segments [24]. As a result, pinpointing to the gene or the genes that are putative driver alterations in each recurrent amplification is a conundrum and represents a more difficult task to identify with certainty compared with recurrent deletions [27]. Several recurrent amplifications in cancer are not primary-site specific, but they are observed in a spectrum of primary cancer types. The amplification of locus 3q26 is a squamous cancers-specific copy-number alteration and is observed in one out of six squamous cervical carcinomas and in an even higher frequency in squamous lung and esophageal cancers [6,13,14,15,16]. Cervical squamous carcinomas with 3q26 amplifications tend to present at a younger age than non-amplified cancers and display a paucity of mutations in TP53. In contrast, additional copy-number gains in segments of chromosomes 19q, 20q, and 8q are more common in 3q26-amplified cervical squamous carcinomas than non-amplified ones. TMB and aneuploidy scores show no significant differences between the two groups of 3q26-amplified and non-amplified cancers, and the same is true for prognosis.

Among the cancer-associated oncogenes located at 3q26 and amplified in squamous carcinomas of the uterine cervix, *PIK3CA*, encoding for the alpha catalytic sub-unit of kinase PI3K, is a prime candidate for being a driver alteration. This is supported by the presence of activated mutations in *PIK3CA* as the most common mutations in the disease with a partially overlapping incidence, suggesting that mutations and amplifications are alternative means that cervical squamous cancer cells use to up-regulate the activity of the oncogenic kinase. However, when both mutations and amplifications were taken into consideration, cervical cancers with *PIK3CA* molecular lesions had a trend for a better disease-free survival than cancers without such lesions, suggesting that the subset of cervical cancers with *PIK3CA* alterations may harbor additional required alterations conferring a less aggressive course [7]. In this regard, *PIK3CA* mutant cancers have a higher TMB and higher rate of mutations in DDR- and MSI-associated genes, while 3q26-amplified cancers display a paucity of *TP53* mutations, as shown above.

*DCUN1D1* (Defective in Cullin Neddylation 1 domain containing 1, alternatively named SCCRO-Squamous Cell Carcinoma-Related Oncogene) is another cancer-associated gene located at 3q26 [28,29]. DCUN1D1 serves as a ligase for the ubiquitin-like protein NEDD8 (Neural precursor cell Expressed, Developmentally Down-Regulated 8). NEDDylation promotes assembly of cullin-type ubiquitin ligases, and as a result, DCUN1D1 regulates ubiquitination of a wide range of substrates that are specific targets of cullin-RING ligases (CRLs). DCUN1D also possesses a ubiquitin binding domain that senses increased ubiquitinated proteins and negatively regulates NEDDylation activity [30]. Decreased NEDD8 attachment interferes with assembly of CRL-type ligases and ubiquitination of client proteins. Among targets regulated by ubiquitination through CRLs are important proteins participating in cell-cycle regulation and cancer-associated signal transduction [31]. In addition, proteins ubiquitinated by other ligases, for example, p53, may be sensed by DCUN1D1 as a signal to down-regulate CRL-mediated ubiquitination. The implications of this down-regulation for processes involving CRL-associated ubiquitination would be broad and far reaching for cancer cells with DCUN1D1 amplifications, including cervical squamous carcinomas. Interestingly, a homologous to DCUN1D1 gene, *DCUN1D5* is located at 11q22 and is amplified in 8.1% of samples in cervical cancer TCGA cohort. 11q22 is the second most commonly amplified segment in cervical squamous carcinomas (up to 11% of cases), and it also contains apoptosis regulators BIRC2 and BIRC3, amplified in 11.3% of cases, as well as several matrix metalloproteinases (MMPs) and the progesterone receptor gene, *PGR*. 11q22 amplifications tend to be more prevalent in 3q26 non-amplified cervical cancers, as shown here, and thus amplification of the two chromosomal segments may be alternative means of cancer cells to up-regulate NEDDylation ligase homologs.

*TBL1XR1* (Transducin beta like 1 X-linked Receptor 1), member of WD40 repeat-containing family, is a component of both nuclear receptor corepressor (N-CoR) and histone deacetylase 3 (HDAC 3) complexes [32]. TBL1XR1 is required for transcriptional activation by a variety of transcription factors. It promotes activation of WNT signaling through interaction with β-catenin, leading to occupancy of target promoters [33]. TBL1XR1 regulates the transcription of ligand VEGF-C in esophageal squamous cancer cells [34]. Squamous cervical cancers are sensitive to VEGF inhibition, and the monoclonal antibody VEGF inhibitor bevacizumab is effective in combination with chemotherapy in a subset of patients with metastatic disease [5]. It would be interesting to investigate whether 3q26 amplification endows cancer cells that bear it with sensitivity to anti-angiogenic agents. In addition, TBL1XR1 is involved in cancer stem cell maintenance through activation of the MEK and PI3K/Akt pathways with a mechanism that involves the stem cell factor SOX2, also a 3q26 gene [35].

The stem cell core transcription factor SOX2 is one of the stemness transcription factors, which can reprogram differentiated cells to stem cells [36]. SOX2 expression as well as expression of the other stem cell factors POU5F1 (also called Oct-4) and NANOG is associated with worse recurrence outcomes in localized cervical cancer patients treated with chemoradiation, suggesting that stemness is a resistance mechanism impeding treatment success in these patients [37]. SOX2 protein abundance derived from amplification of its locus is common in squamous carcinogenesis and is used clinically to confirm a squamous profile in evaluation of lung cancers [38]. A co-operative effect with PI3K signaling has been observed in squamous lung cancers from early stages of neoplastic development [39].

*PRKCI* (Protein Kinase C λ/ι) gene encodes for an atypical protein kinase C, which plays a role in cell polarity. Maintenance of cell polarity in epithelial cells is part of their identity and constitutes a tumor-suppressing mechanism [40]. In contrast, PKCλ/ι has a pro-carcinogenic effect by phosphorylating and activating SOX2, an effect that may be more relevant in 3q26-amplified cancers [41]. The protein localizes in intercellular junctions and in the cytoplasm in normal cervical epithelial cells, but it is often mis-localized in the nucleus in cervical carcinoma cells [42]. Overexpression of the atypical kinase in in situ cervical neoplasia cases was associated with progression to invasive cervical carcinomas, suggesting that the amplification of the gene may be a contributing factor for progression from pre-invasive to invasive stage of cervical carcinogenesis [43].

The two remaining cancer associated genes amplified from 3q26—*MECOM*, encoding for the transcription factor EVI1, and *TERC*, encoding for the human telomerase RNA component—are not correspondingly overexpressed at the mRNA level in 3q26-amplified squamous cell cancers. As a result, they are less likely to be the driver oncogenes in these cancers, despite mechanistic associations of their protein products with putative cancer-promoting processes. Moreover, increased copy numbers, which appear to be associated with progression of cervical cancers from pre-invasive to invasive stages and promotion of HPV integration to cell genome, could be due to an underlying defect leading to increased aneuploidy [44].

The complete absence of *TP53* mutations in 3q26-amplified cervical squamous carcinomas implies that all amplified cancers are HPV-related in contrast with a small percentage of 3q26-non-amplified cancers that are *TP53* mutated and thus possibly non-HPV related. The presence of HPV genome in transformed cells allows for inactivation of p53 through proteasome-mediated degradation facilitated by HPV E6 protein and thus negates the need for mutations in *TP53* gene. In addition, given the association of HPV infection with sexual transmission, 3q26-amplified cancers are more commonly presenting than 3q26 non-amplified cancers in ages younger than 65.

An additional important assertion derived from the current study through interrogation of the cervical cancer cell line panel in the GDSC database is that none of several commonly used cell lines of cervical cancer contains amplifications of 3q26 and thus would not be accurate models for this subset of squamous carcinomas. Cervical cell lines display a dependence in the presence of ubiquitination-related protein as deduced by a CRISPR array. This vulnerability could be explored further therapeutically. 3q26-amplified cervical cancers are theoretically a good candidate to consider, as they possess amplifications of *DCUN1D1*, a regulator of CRLs-mediated ubiquitination.

As an alternative for the pre-clinical study of 3q26-amplified cancers, cell lines established directly from cervical cancer patients with tumors bearing the amplified segment may be preferable for in vitro and in vivo models. Preclinical evaluations with samples derived from patient tumors and bearing the specific molecular lesion of interest, in this case the amplification of 3q26, are the first step for development of rational targeted therapies based on potential vulnerabilities attributed by the presence of the specific molecular defect. These studies may also shed light to the genes from 3q26 that are key drivers of cervical carcinogenesis. Driver genes and their product proteins will constitute candidate targets for effective rational therapies.

## 5. Conclusions

Amplification of 3q26 locus characterizes a sub-set of squamous cervical carcinomas with specific characteristics and may underline specific pathogenesis. Several amplified genes from the locus may contribute to cervical carcinogenesis.

## Figures and Tables

**Table 1:** Amplified genes from loci 3q26.2 to 3q29 listed in OncoKB in TCGA cervical squamous cell carcinoma cohort (*n* = 247) and in subsets with *PIK3CA* (*n* = 39). The subset of cervical squamous cell carcinomas with *MECOM* (*n* = 42) and *TP63* amplifications (*n* = 44) are also presented as examples of overlap of amplifications in more aminoterminal (3q26.2: *MECOM*) and carboxyterminal regions (3q28: *TP63*).

Locus	Gene	Overall Cases (%)	In *PIK3CA* Amplified (%)	In *MECOM* Amplified (%)	In *TP63* Amplified (%)
3q29	*TFRC*	38 (15.4)	34 (87.2)	34 (81)	36 (81.8)
3q28–29	*FGF12*	37 (15)	34 (87.2)	34 (81)	36 (81.8)
3q28	*TP63*	44 (17.8)	35 (89.7)	34 (81)	44 (100)
3q27–28	*LPP*	38 (15.4)	36 (92.3)	35 (83.3)	37 (84.1)
3q27.3	*EIF4A2*	37 (15)	36 (92.3)	35 (83.3)	36 (81.8)
	*BCL6*	36 (14.6)	35 (89.7)	34 (81)	36 (81.8)
3q27.2	*MAP3K13*	36 (14.6)	36 (92.3)	35 (83.3)	35 (79.5)
	*ETV5*	37 (15)	37 (94.9)	36 (85.7)	35 (79.5)
3q27.1	*KLHL6*	37 (15)	37 (94.9)	35 (83.3)	35 (79.5)
3q26.33	*SOX2*	38 (15.4)	38 (97.4)	36 (85.7)	35 (79.5)
	*DCUN1D1*	38 (15.4)	38 (97.4)	36 (85.7)	35 (79.5)
3q26.32	*PIK3CA*	39 (15.8)	39 (100)	37 (88.1)	35 (79.5)
	*TBL1XR1*	40 (16.2)	39 (100)	38 (90.5)	35 (79.5)
3q26.2	*TERC*	40 (16.2)	37 (94.9)	40 (95.2)	34 (77.3)
	*PRKCI*	41 (16.6)	37 (94.9)	40 (95.2)	34 (77.3)
	*MECOM*	42 (17)	37 (94.9)	42 (100)	34 (77.3)

**Table 2:** Percentage of amplification of OncoKB listed genes at 3q26–29 in different cancers. Data are from TCGA cohorts. GE: Gastroesophageal.

Locus	Gene	Cervical Squamous (*n* = 247)	Cervical Adenocarcinomas (*n* = 46)	Head and Neck Cancer (*n* = 517)	Lung Squamous Carcinomas (*n* = 487)	Lung Adenocarcinomas (*n* = 511)	Esophageal Squamous Carcinomas (*n* = 95)	GE Junction Adenocarcinomas (*n* = 87)
3q29	*TFRC*	15.4%	8.7%	13%	30%	2%	28.4%	8%
3q28–29	*FGF12*	15%	8.7%	13.2%	30%	2%	27.4%	5.7%
3q28	*TP63*	17.8%	8.7%	16.1%	31.6%	2%	33.7%	5.7%
3q27–28	*LPP*	15.4%	8.7%	14.5%	31.8%	1.8%	28.4%	9.2%
3q27.3	*EIF4A2*	15%	8.7%	13.9%	31.8%	1.8%	25.3%	4.6%
	*BCL6*	14.6%	8.7%	13.9%	31%	1.6%	25.3%	5.7%
3q27.2	*MAP3K13*	14.6%	8.7%	14.3%	35.5%	2%	24.2%	4.6%
	*ETV5*	15%	8.7%	14.1%	33.5%	1.8%	25.3%	4.6%
3q27.1	*KLHL6*	15%	8.7%	15.1%	39%	2%	25.3%	4.6%
3q26.33	*SOX2*	15.4%	8.7%	15.7%	39.8%	2%	28.4%	3.4%
	*DCUN1D1*	15.4%	8.7%	15.5%	40%	2%	26.3%	3.4%
3q26.32	*PIK3CA*	15.8%	10.9%	15.7%	37.8%	1.8%	29.5%	4.6%
	*TBL1XR1*	16.2%	8.7%	15.1%	36.8%	2%	30.5%	6.9%
3q26.2	*TERC*	16.2%	10.9%	13%	35.5%	2.7%	29.5%	10.3%
	*PRKCI*	16.6%	10.9%	13.2%	35.9%	2.7%	28.4%	10.3%
	*MECOM*	17%	10.9%	13.2%	35.7%	2.7%	28.4%	10.3%

**Table 3:** Percentage of cases with mRNA expression z scores above 2 compared to diploid samples in all (*n* = 248) and *PIK3CA* amplified (*n* = 39) cervical squamous carcinomas. Data are from TCGA.

Gene	Samples with z > 2 Whole Series (%)	Samples with z > 2 in *PIK3CA* Amplified (%)
*SOX2*	41 (16.5%)	16 (41%)
*DCUN1D1*	106 (42.7%)	25 (64.1%)
*PIK3CA*	98 (39.5%)	25 (64.1%)
*TBL1XR1*	104 (41.9%)	31 (79.5%)
*TERC*	7 (2.8%)	0
*PRKCI*	76 (30.6%)	21 (53.8%)
*MECOM*	4 (1.6%)	0

**Table 4:** Top 8 gene dependencies enriched in cervical squamous context in DepMap. * denotes that the gene was also significant in the CRISPR (Avana) Public 21Q1 dataset, albeit with a higher *p*-value. Data are from DepMap portal.

Gene	Alternative Name-Function	Dataset	T Statistic	*p*-Value
*ZER1*	Ubquitin ligase involved in meiosis	CRISPR (combined) *	−11.8	4 × 10^−30^
*UBE3A*	E6-AP	CRISPR (combined) *	−11.1	3.36 × 10^−27^
*FBXL5*	F-box component of SCF ligases	CRISPR (combined) *	−5.06	4.97 × 10^−7^
*GEN1*	Holliday junction 5′ endonuclease	CRISPR (combined) *	−4.5	7.54 × 10^−6^
*RNF145*	Ring finger ligase	CRISPR (combined) *	4.27	2.1 × 10^−5^
*RAB39B*	Rab family GTPase vesicle trafficking	CRISPR (combined)	−4.09	4.75 × 10^−5^
*SKP2*	FBXL1, F-box component of SCF ligases	CRISPR (combined) *	−3.94	8.76 × 10^−5^
*CUL2*	Cullin component of SCF ligases, E7 interacting	CRISPR (combined)	−3.86	0.000122

## Data Availability

No additional data besides the ones presented in the article are available.
